# Deep Learning Algorithms in the Diagnosis of Basal Cell Carcinoma Using Dermatoscopy: Systematic Review and Meta-Analysis

**DOI:** 10.2196/73541

**Published:** 2025-10-03

**Authors:** Huasheng Liu, Guangqian Shang, Qianqian Shan

**Affiliations:** 1 Department of Burn Plastic and Cosmetic Surgery Beijing Jishuitan Hospital Liaocheng Hospital Liaocheng City China; 2 Department of Burn Plastic and Cosmetic Surgery Liaocheng People's Hospital Liaocheng City China; 3 Department of Medical cosmetic Chiping District People's Hospital Liaocheng City China; 4 Department of Gynecology and Obstetric Liaocheng People's Hospital Liaocheng City China

**Keywords:** deep learning algorithms, dermatoscopy, basal cell carcinoma, meta-analysis, artificial intelligence, AI

## Abstract

**Background:**

In recent years, deep learning algorithms based on dermatoscopy have shown great potential in diagnosing basal cell carcinoma (BCC). However, the diagnostic performance of deep learning algorithms remains controversial.

**Objective:**

This meta-analysis evaluates the diagnostic performance of deep learning algorithms based on dermatoscopy in detecting BCC.

**Methods:**

An extensive search in PubMed, Embase, and Web of Science databases was conducted to locate pertinent studies published until November 4, 2024. This meta-analysis included articles that reported the diagnostic performance of deep learning algorithms based on dermatoscopy for detecting BCC. The quality and risk of bias in the included studies were assessed using the modified Quality Assessment of Diagnostic Accuracy Studies 2 tool. A bivariate random-effects model was used to calculate the pooled sensitivity and specificity, both with 95% CIs.

**Results:**

Of the 1941 studies identified, 15 (0.77%) were included (internal validation sets of 32,069 patients or images; external validation sets of 200 patients or images). For dermatoscopy-based deep learning algorithms, the pooled sensitivity, specificity, and area under the curve (AUC) were 0.96 (95% CI 0.93-0.98), 0.98 (95% CI 0.96-0.99), and 0.99 (95% CI 0.98-1.00). For dermatologists’ diagnoses, the sensitivity, specificity, and AUC were 0.75 (95% CI 0.66-0.82), 0.97 (95% CI 0.95-0.98), and 0.96 (95% CI 0.94-0.98). The results showed that dermatoscopy-based deep learning algorithms had a higher AUC than dermatologists’ performance when using internal validation datasets (*z*=2.63; *P*=.008).

**Conclusions:**

This meta-analysis suggests that deep learning algorithms based on dermatoscopy exhibit strong diagnostic performance for detecting BCC. However, the retrospective design of many included studies and variations in reference standards may restrict the generalizability of these findings. The models evaluated in the included studies generally showed improved performance over that of dermatologists in classifying dermatoscopic images of BCC using internal validation datasets, highlighting their potential to support future diagnoses. However, performance on internal validation datasets does not necessarily translate well to external validation datasets. Additional external validation of these results is necessary to enhance the application of deep learning in dermatological diagnostics.

**Trial Registration:**

PROSPERO International Prospective Register of Systematic Reviews CRD42025633947; https://www.crd.york.ac.uk/PROSPERO/view/CRD42025633947

## Introduction

### Background

Basal cell carcinoma (BCC) is the most common type of skin cancer, accounting for approximately 80% of all nonmelanoma skin cancers worldwide [1]. Its incidence has been increasing annually, with a global estimated growth rate of 3% to 10% [2,3]. Although BCC rarely metastasizes, its local invasiveness can lead to significant patient distress, cosmetic damage, and health care burdens [4]. Early and accurate diagnosis is crucial to ensure timely intervention, reduce the risk of complications, and improve patient outcomes. Despite advances in diagnostic technologies, challenges remain in achieving high diagnostic accuracy and consistency.

Dermatoscopy is a widely used tool for diagnosing BCC [5]. It is a noninvasive imaging technique that enhances the visualization of subsurface structures of skin lesions through magnification and illumination [6]. Dermatoscopy facilitates improved observation of skin lesions, allowing for a more accurate assessment of their characteristics [5]. This method uses epiluminescence, which involves illuminating the skin surface with light that passes through a transparent medium, minimizing surface reflection and enhancing the visualization of the lesion’s detailed features [7]. However, its diagnostic accuracy can vary due to interobserver or intraobserver differences, and human observers typically rely on qualitative assessments of morphological features [8-10]. In addition, although capture of dermatoscopy using digital images produces a large amount of quantitative data, much of this information remains underused due to the limitations of human visual perception [10]. These challenges highlight the need for more robust and objective diagnostic methods to improve the detection and classification of BCC.

In recent years, deep learning algorithms based on dermatoscopy have shown great potential in diagnosing BCC. These algorithms use artificial intelligence (AI) to analyze complex imaging data, identifying patterns and features that are difficult for the human eye to detect [11]. However, the diagnostic performance of deep learning algorithms remains controversial. Due to the “black box” nature of deep learning models, it is unclear which image features are deemed most important by the algorithms [12]. Some studies report varying levels of accuracy, sensitivity, and specificity, raising concerns about the universality and reliability of deep learning algorithms across different datasets and clinical settings [11]. Furthermore, the relative diagnostic performance of deep learning algorithms versus human experts remains a contentious issue, with conflicting findings in the literature [13,14]. These controversies indicate the need for a comprehensive evaluation of the effectiveness of deep learning–based BCC diagnostic methods.

### Objectives

The aim of this meta-analysis was to assess the diagnostic performance of deep learning algorithms based on dermatoscopy in detecting BCC and compare them with the diagnostic performance of human experts.

## Methods

This meta-analysis was conducted in strict adherence to the guidelines outlined in the PRISMA-DTA (Preferred Reporting Items for Systematic Reviews and Meta-Analyses extension for Diagnostic Test Accuracy) checklist [15]. In addition, the protocol for this study was officially registered in PROSPERO (CRD42025633947).

### Search Strategy

We conducted a literature search in 3 databases (PubMed, Embase, and Web of Science) with the search cutoff date of November 4, 2024. A second search was conducted in January 2025 to supplement with newly published studies. The search strategy included 2 groups of keywords: the first group related to AI terms (eg, AI, machine learning, deep learning), and the second group related to target terms (eg, skin neoplasms, BCC, nonmelanoma skin cancer). We used a combination of free terms and MeSH (Medical Subject Headings) for the search strategy, with detailed information provided in Multimedia Appendix 1. In addition, we searched the reference lists of the studies included in the final selection for relevant literature.

### Inclusion and Exclusion Criteria

The selection of studies was meticulously guided by the population, intervention, comparison, outcome, and study design framework, shown in Textbox 1, to ensure methodological rigor.

The study selection process involved systematic steps, with specific exclusion criteria applied at each stage. Initially, studies identified from databases were screened, and after removing duplicates, studies involving animals or nonoriginal research work (such as reviews, case reports, conference abstracts, and meta-analyses) were excluded from the initial screening. The remaining records were then assessed, and a comprehensive review of the full-text articles was conducted, leading to the exclusion of studies that lacked essential information (including true positives [TPs], false positives [FPs], false negatives [FNs], and true negatives [TNs]). Studies that did not focus specifically on BCC detection or those that did not use dermatoscopy AI models were also excluded. In addition, non–English-language studies were removed due to accessibility concerns. Finally, studies using non–deep learning AI algorithms were excluded to maintain consistency in assessing advanced computational methods.

**Inclusion criteria based on the population, intervention, comparison, outcome, and study design framework.**

**Inclusion criteria**

- Population: patients undergoing basal cell carcinoma detection
- Intervention: studies using deep learning models applied to dermatoscopy images
- Comparison: performance of models compared against that of dermatologists; studies with no control group were also acceptable
- Outcome: metrics assessed including sensitivity, specificity, and area under the curve
- Study design: both retrospective and prospective studies
- Additional criteria: studies published in the English language

### Quality Assessment

To rigorously assess the quality of the included studies, we modified the Quality Assessment of Diagnostic Accuracy Studies 2 (QUADAS-2) tool [16] by replacing some irrelevant criteria with more applicable risk assessment standards for predictive models [17]. The modified QUADAS-2 tool includes 4 fundamental domains: patient selection, index test (AI algorithm), reference standard (RS), and analysis. We assessed the risk of bias for these 4 domains and evaluated the applicability concern for the first 3 domains. Two reviewers independently used the modified QUADAS-2 tool to assess the risk of bias in the included studies, and any disagreements between reviewers were resolved through discussion. The risk of bias was rated as high, low, or unclear.

### Data Extraction

Two reviewers (HL and GS) independently conducted an initial screening of the titles and abstracts of the remaining articles to determine potential eligibility. Any disagreements were resolved through arbitration by a third reviewer (QS) to reach a consensus. The extracted data included the first author’s name, year of publication, study design, study country, target condition, image type, RS, whether the data were open access, patients or images per set (training, internal validation or test sets, and external validation or test sets), diagnostic model, and diagnostic performance indicators (TPs, FPs, FNs, and TNs).

As most studies did not provide diagnostic contingency tables, we used two strategies to construct the diagnostic 2 × 2 tables: (1) using sensitivity, specificity, the number of positives according to the RS, and the total number of cases; and (2) conducting receiver operating characteristic (ROC) curve analysis extracting the best sensitivity and specificity values based on the optimal Youden index. However, inferring data through ROC curve analysis can introduce potential errors. The optimal cutoff value may not accurately reflect real-world diagnostic performance, leading to misclassification of cases and affecting the calculated values of TPs, FPs, FNs, and TNs.

### Outcome Measures

The primary outcome measures were sensitivity, specificity, and area under the curve (AUC) for the internal validation set, the external validation set, and dermatologists’ diagnoses. Sensitivity (also known as recall or TP rate) measures the probability that the deep learning model will correctly identify BCC, calculated as TP/(TP+FN). Specificity (also known as the TN rate) reflects the probability that the deep learning model will correctly identify non-BCC, calculated as TN/(TN+FP). The area under the ROC curve, known as AUC, is an overall measure of how effectively the model differentiates between positive and negative instances. As part of the revision, we excluded whole-slide imaging studies and focused only on dermatoscopy-based studies based on reviewer feedback. This change was made to allow for a more consistent and focused analysis.

For studies that provided multiple contingency tables based on different datasets, we assumed these to be independent and extracted data from all available tables. In addition, for studies that used multiple deep learning models, we only included the model with the highest AUC from the internal and external validation sets. Furthermore, when comparing our results with those of dermatologists, we used a non–head-to-head comparison as there were only 5 datasets (5 datasets exclusively contain diagnostic data from dermatologists) derived from the included studies, whereas the AI data comprised 16 datasets (16 datasets solely include diagnostic data from AI models). It is also important to note that, due to limitations in the study data, we did not categorize different dermatologists by experience level, such as junior and senior.

### Statistical Analysis

In this study, a bivariate random-effects model was used for the meta-analysis to evaluate the diagnostic performance of deep learning models in diagnosing BCC based on dermatoscopy images. The overall sensitivity and specificity for the internal validation set, the external validation set, and dermatologists’ or pathologists’ diagnoses were summarized separately. Forest plots were used to visually display the combined sensitivity and specificity, and summary ROC curves were drawn to provide combined estimates along with their 95% CIs and prediction intervals. The heterogeneity between studies was assessed using the Higgins *I*^2^ statistic, with *I*^2^ values of 25%, 50%, and 75% corresponding to low, moderate, and high heterogeneity, respectively. For internal validation sets with sample sizes of >10, meta-regression analysis was conducted for significant heterogeneity (*I*^2^>50%) to explore potential sources of heterogeneity. The variables included in the meta-regression were AI method, RS, type of internal validation, and image magnification. Furthermore, univariate subgroup analyses were conducted for the aforementioned variables, and the likelihood ratio test was used to assess statistical differences between subgroups.

The Deeks funnel plot asymmetry test was used to evaluate publication bias. Statistical analyses were conducted using the *midas* and *metadta* packages in Stata (version 15.1; StataCorp), and the risk-of-bias assessment for study quality was conducted using the Cochrane Collaboration’s RevMan software (version 5.4). All statistical tests were 2-sided, with *P*<.05 considered statistically significant.

## Results

### Study Selection

**Figure 1:**
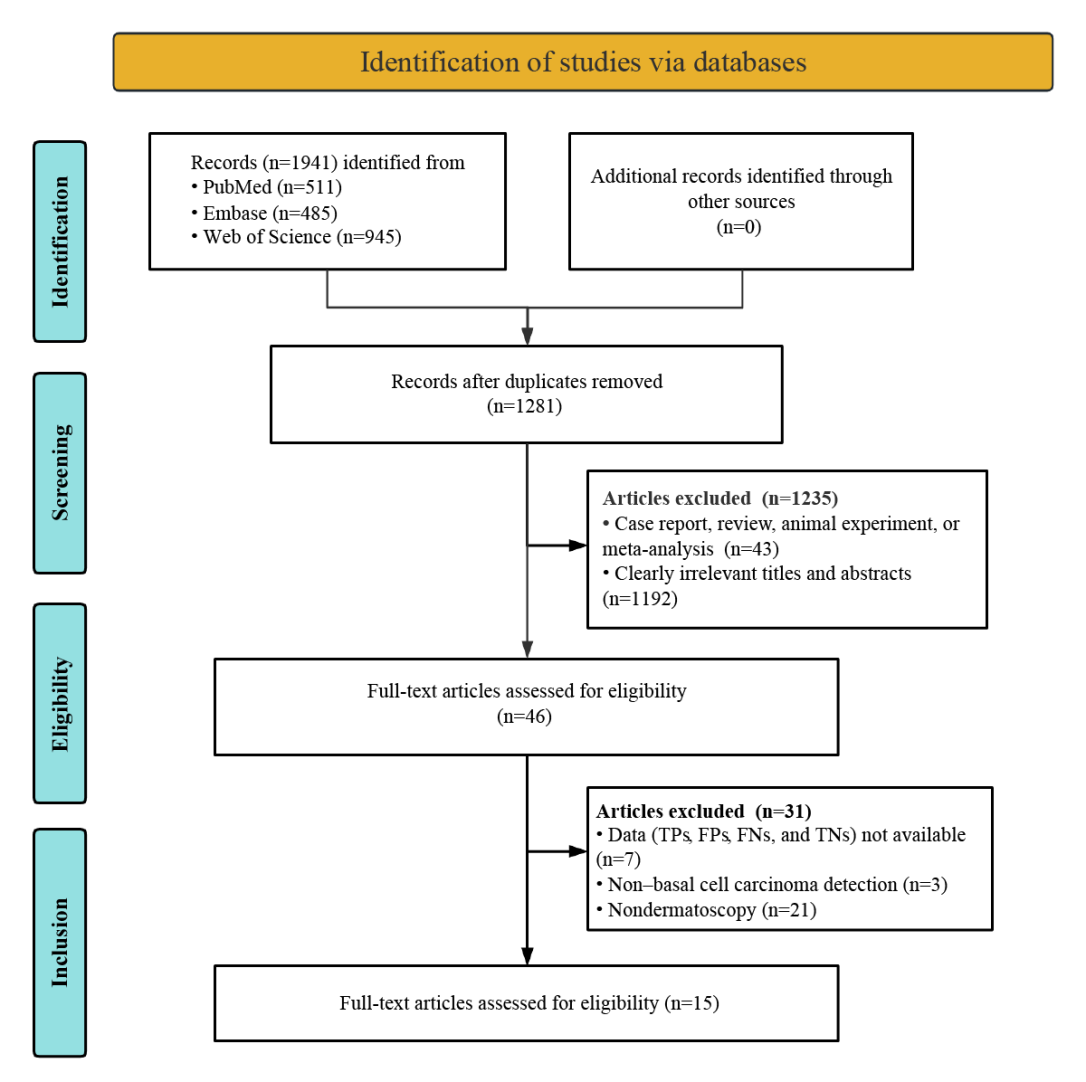
PRISMA (Preferred Reporting Items for Systematic Reviews and Meta-Analyses) flow diagram illustrating the study selection process. FN: false negative; FP: false positive; TN: true negative; TP: true positive.

The initial database search yielded 1941 potentially relevant articles. Of these 1941 articles, 1281 (66%) proceeded to preliminary screening after removing 660 (34%) duplicates. After strictly applying the inclusion criteria, of the 1281 articles preliminarily screened, 1235 (96.41%) were excluded. After conducting a thorough review of the 46 full texts, 7 (15%) studies were excluded for having insufficient or incomplete diagnostic data (TPs, FPs, FNs, and TNs), 3 (7%) studies were removed because they did not detect BCC, and 21 (46%) studies were excluded due to nondermatoscopy methods. In the end, 15 studies assessing the diagnostic capabilities of deep learning models were included in the meta-analysis [18-32]. The process of study selection is thoroughly detailed in accordance with the standardized PRISMA (Preferred Reporting Items for Systematic Reviews and Meta-Analyses) flowchart illustrated in Figure 1.

### Study Description and Quality Assessment

A total of 15 studies based on dermatoscopy deep learning algorithms were included [18-32]. Of these 15 studies, 14 (93%) included internal validation sets comprising a total of 32,069 patients or images (range 50-13,603), and 1 (7%) included an external validation set with a total of 200 patients or images [22]. In total, 27% (4/15) of the studies assessed the diagnostic performance of dermatologists [18,22,27,32]. Of the 15 included studies, 6 (40%) were funded through sponsorship. The studies were published between 2011 and 2024. Of the 15 studies included in the meta-analysis, 14 (93%) were retrospective studies, and 1 (7%) was a prospective study [18,27]. A total of 13% (2/15) of the studies used only histopathology as the RS [19,27], whereas 87% (13/15) of the studies used histopathology with expert consensus or clinical follow-up. The most commonly used optimal deep learning algorithm was convolutional neural network (CNN; 12/15, 80%). A summary detailing the study and patient characteristics and technical features can be found in Tables 1 and 2 and Multimedia Appendices 2 [18,22,27,32] and 3 [18-32].

**Table 1:** Study and patient characteristics.

Study	Year	Country	Study design	Target condition	Reference standard	Open access data	Patients or images per set		
							Training	Internal validation or test sets	External validation or test sets
Wang et al [18]	2020	China	Prospective	BCC^a^	Histopathology and expert consensus	No	7192	70	NR^b^
Kharazmi et al [19]	2018	Canada	Retrospective	BCC	Histopathology	No	449	450	NR
Maurya et al [20]	2024	United States	Retrospective	BCC	Histopathology and expert consensus	Yes	2000	395	NR
UdriȘtoiu et al [21]	2020	Romania	Retrospective	BCC	Histopathology, expert consensus, and clinical follow-up	Yes	23,018	1249	NR
Zhu et al [22]	2021	China	Retrospective	BCC	Histopathology and expert consensus	No	13,603	13,603	200
Serrano et al [23]	2022	Spain	Retrospective	BCC	Histopathology, expert consensus, and clinical follow-up	Yes	5484	564	NR
Cheng et al [24]	2011	United States	Retrospective	BCC	Histopathology, expert consensus, and clinical follow-up	No	175	211	NR
Maurya et al [25]	2024	United States	Retrospective	BCC	Histopathology, expert consensus, and clinical follow-up	Yes	1288	395	NR
Radhika and Chandana [26]	2023	India	Retrospective	BCC	Histopathology and expert consensus	Yes	17,731	5066	NR
Maron et al [27]	2019	Germany	Retrospective	BCC	Histopathology	Yes	12,336	300	NR
Naeem et al [28]	2022	Pakistan and South Korea	Retrospective	BCC	Histopathology and expert consensus	Yes	17,731	5066	NR
Ali et al [29]	2023	South Korea	Retrospective	BCC	Histopathology, expert consensus, and clinical follow-up	Yes	5600	400	NR
Panthakkan et al [30]	2022	United Arab Emirates	Retrospective	BCC	Histopathology, expert consensus, and clinical follow-up	Yes	8400	2100	NR
Priyeshkumar et al [31]	2024	India	Retrospective	BCC	Histopathology, expert consensus, and clinical follow-up	Yes	14,700	2100	NR
Minagawa et al [32]	2020	Japan	Retrospective	BCC	Histopathology, expert consensus, and clinical follow-up	Partial	12,848	50/50	NR

^a^BCC: basal cell carcinoma.

^b^NR: not reported.

**Table 2:** Technical aspects of the included studies.

Study	Year	Type of internal validation	DL^a^ model	Optimal DL algorithms	Internal validation or test sets				External validation or test sets			
					TP^b^	FP^c^	FN^d^	TN^e^	TP	FP	FN	TN
Wang et al [18]	2020	10-fold cross-validation	GoogLeNet Inception version 3	CNN^f^	8	0	2	60	NR^g^	NR	NR	NR
Kharazmi et al [19]	2018	Random split test set	SAE^h^ with softmax classifier	Autoencoder	128	18	22	282	NR	NR	NR	NR
Maurya et al [20]	2024	Random split test set	Hybrid model of EfficientNet-B5+random forest classifier	CNN	190	4	5	196	NR	NR	NR	NR
UdriȘtoiu et al [21]	2020	Random split test set	4-layer CNN	CNN	185	11	2	1051	NR	NR	NR	NR
Zhu et al [22]	2021	NR	Modified CNN model based on EfficientNet-B4	CNN	473	275	14	12,841	22	1	3	174
Serrano et al [23]	2022	Random split test set	VGG16 with custom fully connected layers	CNN	302	15	2	245	NR	NR	NR	NR
Cheng et al [24]	2011	Leave-one-out cross-validation	Standard backpropagation neural network	MLP^i^	55	22	4	130	NR	NR	NR	NR
Maurya et al [25]	2024	Random split test set	Hybrid model of EfficientNet-B5+U-Net+random forest classifier	CNN	191	7	4	193	NR	NR	NR	NR
Radhika and Chandana [26]	2023	Random split test set	MSCD-Net^j^ model	CNN	550	95	72	4349	NR	NR	NR	NR
Maron et al [27]	2019	Random split test set	ResNet-50	CNN	52	4	8	236	NR	NR	NR	NR
Naeem et al [28]	2022	Leave-one-out cross-validation	SCDNet^k^ (VGG16+CNN)	CNN	550	95	72	4349	NR	NR	NR	NR
Ali et al [29]	2023	10-fold cross-validation	26-layer CNN model	CNN	197	12	3	188	NR	NR	NR	NR
Panthakkan et al [30]	2022	5-fold cross-validation	Concatenated Xception–ResNet-50 model	CNN	323	8	7	1762	NR	NR	NR	NR
Priyeshkumar et al [31]	2024	Random split test set	Mg-EDCF^l^ model	DCF^m^	296	24	4	1776	NR	NR	NR	NR
Minagawa et al [32]^n^	2020	Random split test set	Inception–ResNet version 2	CNN	9	1	3	37	NR	NR	NR	NR
Minagawa et al [32]^o^	2020	Random split test set	Inception–ResNet version 2	CNN	12	0	0	38	NR	NR	NR	NR

^a^DL: deep learning.

^b^TP: true positive.

^c^FP: false positive.

^d^FN: false negative.

^e^TN: true negative.

^f^CNN: convolutional neural network.

^g^NR: not reported.

^h^SAE: sparse autoencoder.

^i^MLP: multilayer perceptron.

^j^MSCD-Net: multiclass skin cancer detection network.

^k^SCDNet: skin cancer detection classifier network.

^l^Mg-EDCF: multigrained enhanced deep cascaded forest.

^m^DCF: deep cascaded forest.

^n^Shinshu test set.

^o^International Skin Imaging Collaboration test set.

**Figure 2:**
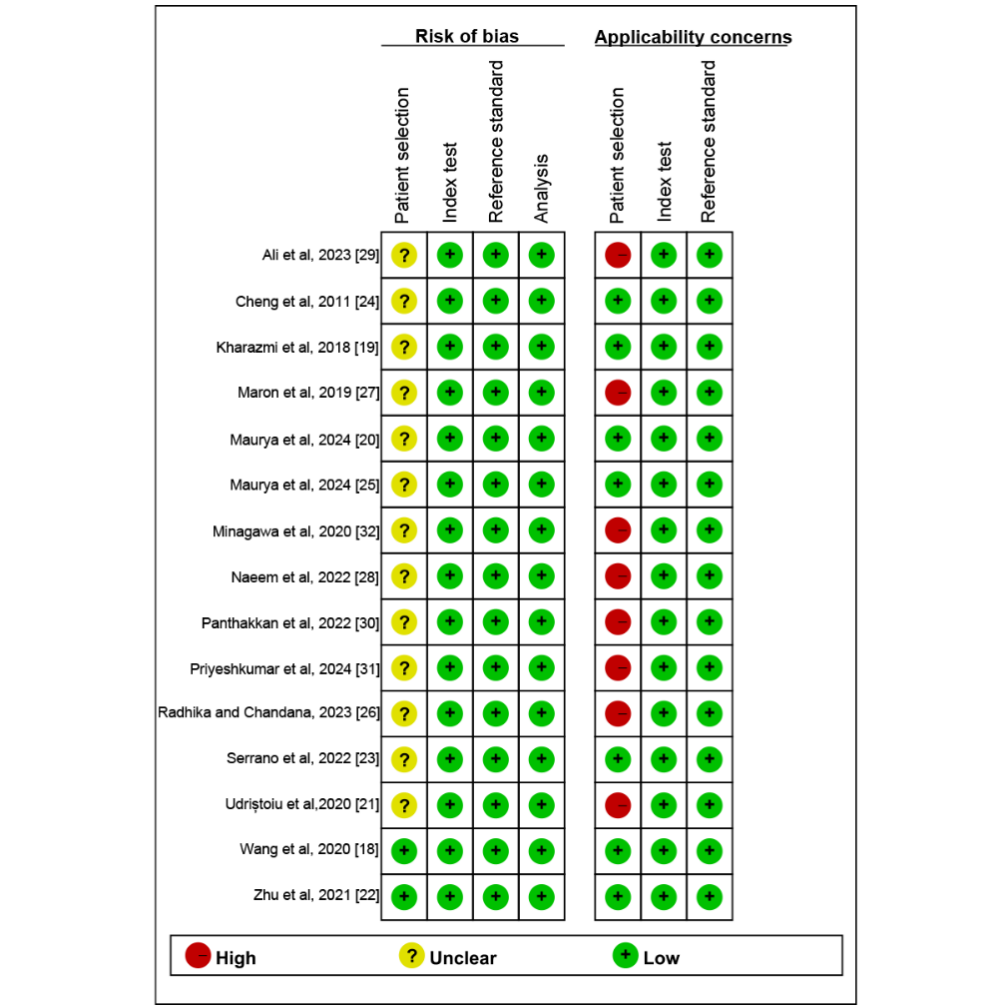
Risk of bias and applicability concerns of the included studies using the modified Quality Assessment of Diagnostic Performance Studies 2 tool [18-32].

Figure 2 [18-32] and Multimedia Appendix 4 [18-32] display the risk-of-bias evaluation conducted using the modified QUADAS-2 tool. Regarding concerns about applicability in patient selection, 53% (8/15) of the studies were also designated as high risk due to their inclusion of patients with other types of malignant skin tumors. No studies were identified as high risk regarding the index test, nor were any studies deemed high risk for the RS. Ultimately, the quality assessment indicated that the studies included in this review were generally considered to have an acceptable quality.

### Diagnostic Performance of Dermatoscopy-Based Deep Learning Algorithms Versus Dermatologists in Detecting BCC

**Figure 3:**
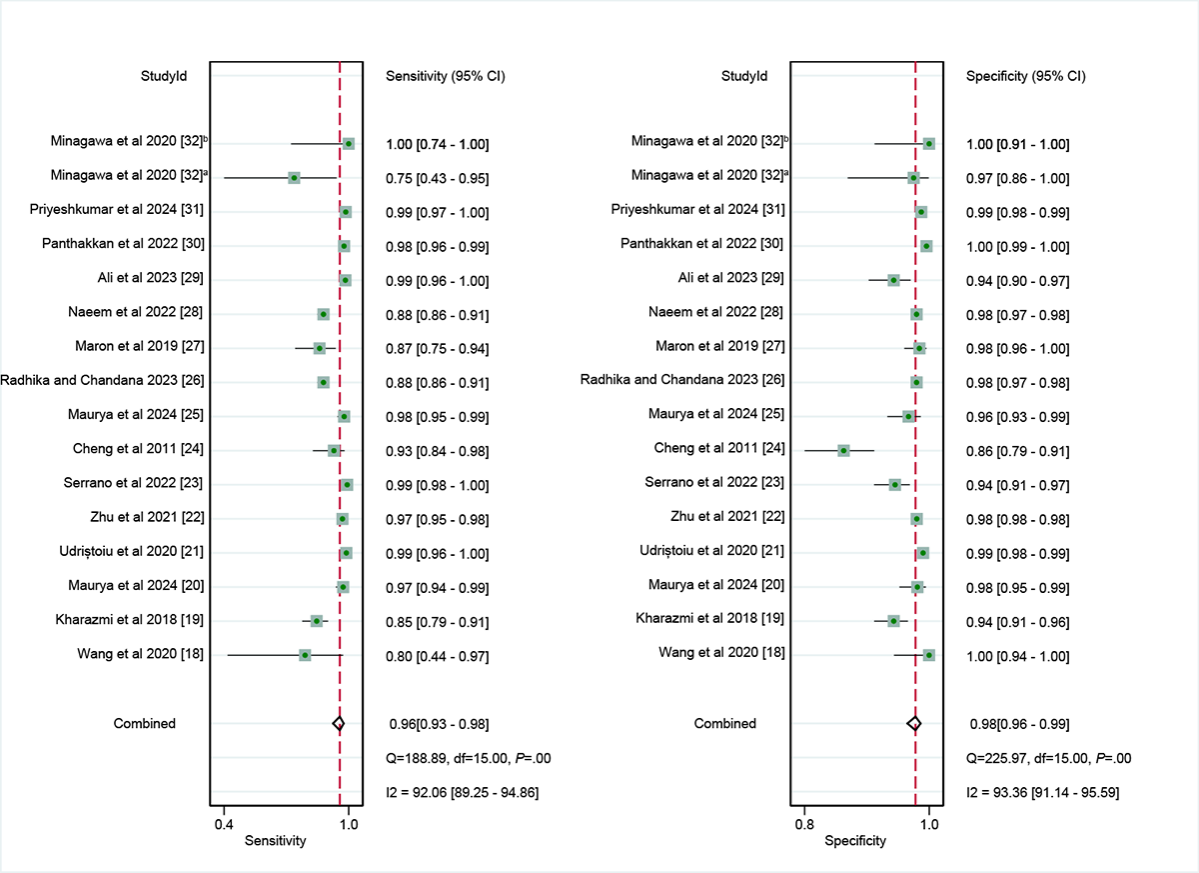
Forest plot of the sensitivity and specificity of deep learning algorithms in the diagnosis of basal cell carcinoma using dermatoscopy on the internal test dataset [18-32]. a: Shinshu test set; b: International Skin Imaging Collaboration test set.

**Figure 4:**
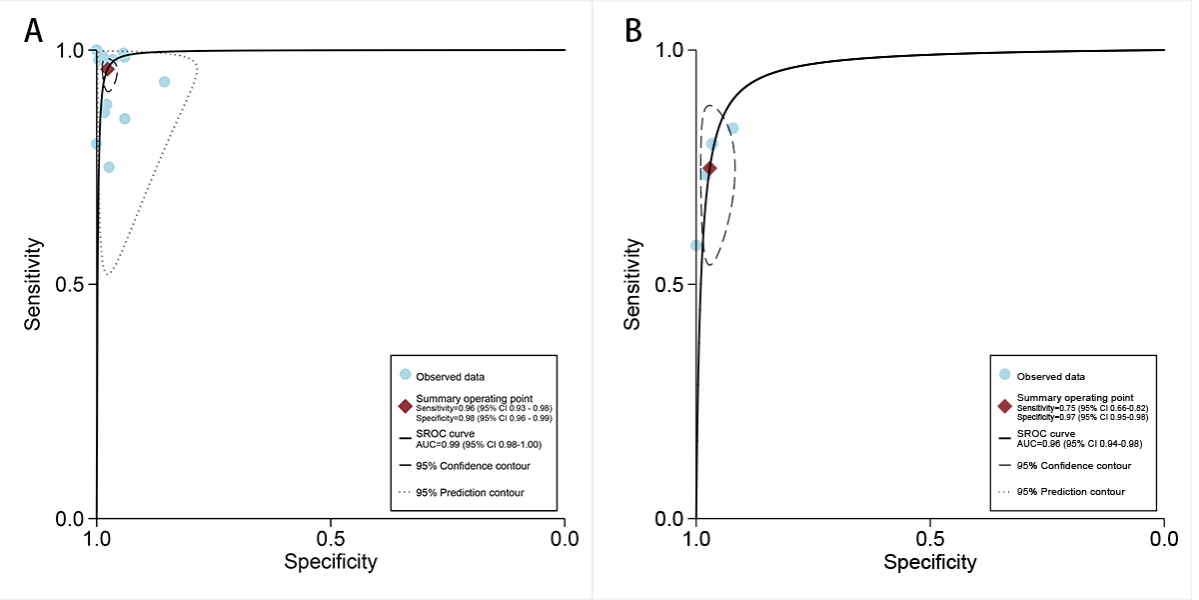
Summary receiver operating characteristic (SROC) curves of deep learning algorithms on the internal validation set (A) and dermatologists’ diagnoses (B) for basal cell carcinoma using dermatoscopy. AUC: area under the curve.

**Figure 5:**
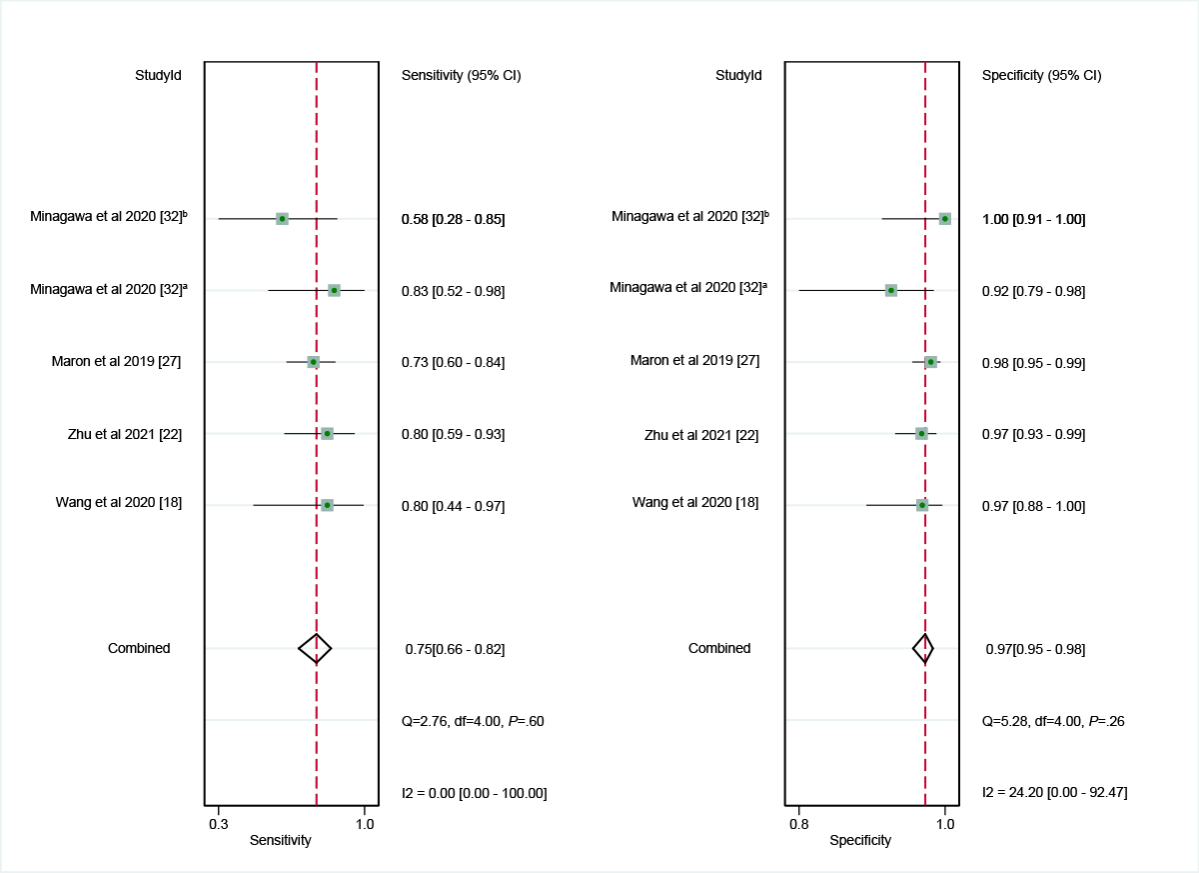
Forest plot of the sensitivity and specificity of dermatologists’ diagnoses of basal cell carcinoma using dermatoscopy [18,22,27,32]. a: Shinshu test set; b: International Skin Imaging Collaboration test set.

In the internal validation dataset, dermatoscopy-based deep learning algorithms demonstrated a sensitivity of 0.96 (95% CI 0.93-0.98) and a specificity of 0.98 (95% CI 0.96-0.99; Figure 3 [18-32]), yielding an AUC of 0.99 (95% CI 0.98-1.00; Figure 4A). In contrast, dermatologists achieved a sensitivity of 0.75 (95% CI 0.66-0.82), with a specificity of 0.97 (95% CI 0.95-0.98; Figure 5 [18,22,27,32]), resulting in an AUC of 0.96 (95% CI 0.94-0.98; Figure 4B). The results showed that dermatoscopy-based deep learning algorithms had a higher AUC than dermatologists when using internal validation datasets (*z*=2.63; *P*=.008).

**Figure 6:**
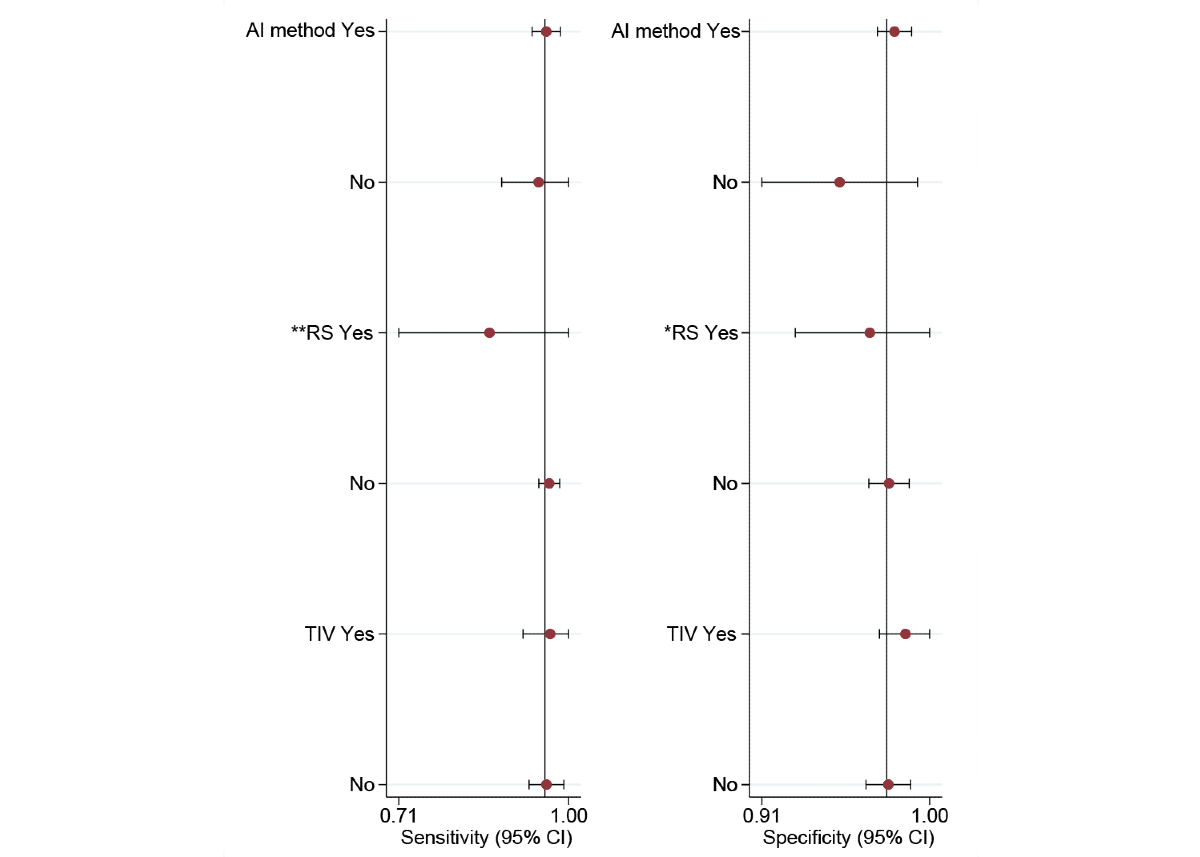
Subgroup analysis and meta-regression analysis for deep learning algorithms in the diagnosis of basal cell carcinoma using dermatoscopy on the internal test dataset（*P<.05; **P<.01; ***P<.001). AI: artificial intelligence; RS: reference standard; TIV: type of internal validation.

For the internal validation set, both sensitivity (τ^2^=1.22; *I*^2^=92.06%) and specificity (τ^2^=0.78; *I*^2^=93.36%) exhibited high heterogeneity. Meta-regression analysis indicated that the heterogeneity was primarily caused by differences in the RS (sensitivity: *P*=.01; specificity: *P*=.05; Figure 6).

### Diagnostic Performance of Deep Learning Algorithms in Detecting BCC in External Validation Sets

In the external validation set for dermatoscopy, only the study conducted by Zhu et al [22] was considered. Their findings indicated that, within this external validation set, dermatoscopy-based deep learning algorithms achieved a sensitivity of 0.88 (95% CI 0.70-0.96) and a specificity of 0.99 (95% CI 0.97-1.00).

### Diagnostic Performance in Subgroup Analyses of Deep Learning Algorithms Based on Dermatoscopy in Detecting BCC

In the AI method subgroup, the sensitivity of CNN and non-CNN methods was 0.96 (95% CI 0.92-0.98; τ^2^=1.29; *I*^2^=51.26%) and 0.95 (95% CI 0.83-0.99; τ^2^=1.11; *I*^2^=79.56%), respectively, with no statistically significant difference (*P*=.74). In addition, the specificity of CNN and non-CNN methods was 0.98 (95% CI 0.97-0.99; τ^2^=0.48; *I*^2^=47.02%) and 0.95 (95% CI 0.89-0.98; τ^2^=1.03; *I*^2^=89.53%), respectively, with no statistically significant difference (*P*=.08).

In the RS subgroup, the sensitivity of only histopathology and histopathology with expert consensus or clinical follow-up was 0.86 (95% CI 0.62-0.96) and 0.97 (95% CI 0.94-0.98; τ^2^=1.13; *I*^2^=47.19%), respectively, with no statistically significant difference (*P*=.07). Correspondingly, the specificity of the 2 RS methods was 0.97 (95% CI 0.89-0.99) and 0.98 (95% CI 0.96-0.99; τ^2^=0.84; *I*^2^=61.30%), respectively, with no statistically significant difference (*P*=.58).

In the type of internal validation subgroup, the sensitivity of fold cross-validation and random split test set was 0.97 (95% CI 0.86-0.99; τ^2^=0.85; *I*^2^=30.44%) and 0.96 (95% CI 0.91-0.98; τ^2^=1.72; *I*^2^=54.89%), respectively, with no statistically significant difference (*P*=.79). Correspondingly, the specificity of the 2 types of internal validation was 0.99 (95% CI 0.96-1.00; τ^2^=6.36; *I*^2^=12.09%) and 0.98 (95% CI 0.96-0.99; τ^2^=0.34; *I*^2^=43.76%), respectively, with no statistically significant difference (*P*=.39; Multimedia Appendix 5).

### Publication Bias

**Figure 7:**
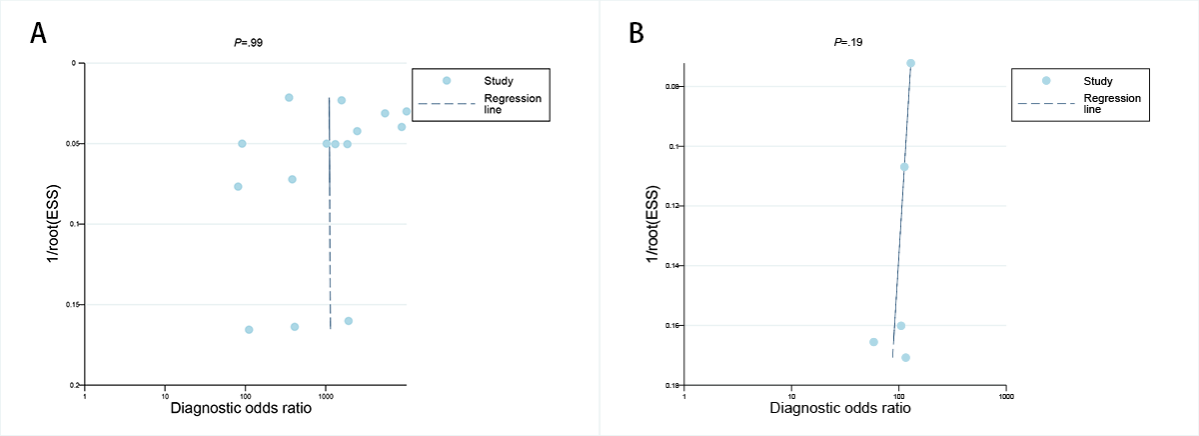
The Deeks funnel plot used to evaluate the publication bias of deep learning algorithms on the internal validation set (A) and dermatologists’ diagnoses (B) for basal cell carcinoma using dermatoscopy (*P*<.05 was considered significant). ESS: effective sample size.

The Deeks funnel plot asymmetry assessment indicated that there was no notable publication bias for the dermatoscopy-based deep learning algorithms in both the internal validation set and among dermatologists (*P*=.99 and .19, respectively; Figures 7A-B).

## Discussion

### Principal Findings

Our meta-analysis shows that dermatoscopy-based deep learning algorithms exhibited exceptional diagnostic performance for BCC, with an AUC value of 0.99, which was significantly higher than that of dermatologists when using internal validation datasets (*P*=.008). The diagnostic potential of deep learning algorithms can be attributed to their ability to process large volumes of high-dimensional data and extract complex patterns [33]. Specifically, in dermatoscopy-based diagnosis, deep learning models use advanced CNNs trained on large datasets to automatically extract features and recognize patterns, surpassing human visual capabilities when performing certain tasks [32]. This advantage is particularly noticeable when detecting microstructural features or changes in lesion images [34]. Furthermore, deep learning algorithms have the potential to be less susceptible to inter- and intraobserver variability [35,36].

### Comparison to Prior Work

Previously, meta-analyses have evaluated deep learning algorithms for the detection of melanoma using dermatoscopic images [14,37]. However, unlike previous meta-analyses, our study only focused on BCC. In addition, to our knowledge, this is the first meta-analysis to evaluate the diagnostic performance of deep learning algorithms for BCC detection using dermatoscopy and compare them with dermatologists’ and pathologists’ diagnoses, providing a broader perspective on AI application across various types of training data. We also conducted the first performance analysis for external validation sets. Our results showed that the diagnostic performance of dermatoscopy-based deep learning algorithms declined in external validation, highlighting the importance of real-world testing when evaluating model reliability and generalizability. Although previous studies have predominantly focused on melanoma or the overall performance of AI in skin cancer diagnosis, our research fills a crucial gap by concentrating on BCC, offering robust evidence on the diagnostic potential of deep learning algorithms in this domain.

### Heterogeneity

The high heterogeneity observed in the studies included in our meta-analysis may have influenced the overall sensitivity and specificity of deep learning algorithms on internal test data. Identifying the specific sources of heterogeneity is crucial for guiding result interpretation of meta-analytic findings in heterogeneous research settings [38]. For the dermatoscopy-based deep learning algorithms, meta-regression analysis identified RS as a major source of heterogeneity. This variation could stem from differences in how the RS was defined and applied across the studies. For example, some studies (2/16, 13%) relied solely on histopathology as the gold standard, whereas others (14/16, 88%) incorporated both clinical and histopathological criteria. The use of different RS definitions may account for some of the observed variability in performance across studies. These methodological inconsistencies highlight the importance of developing standardized protocols in future studies to ensure comparability across research efforts.

### Future Directions

Our results demonstrate that the models in the included studies outperformed dermatologists in classifying dermatoscopic images of BCC using internally validated datasets. Notably, both internal and external validation sets exhibited robust diagnostic accuracy, suggesting that deep learning algorithms have the potential to alleviate the workload of clinicians, reduce misdiagnoses, and prevent delayed or erroneous treatments, thereby preventing adverse outcomes caused by diagnostic delays or incorrect treatments. The implementation of deep learning algorithms in primary care settings could be particularly beneficial for early detection and timely management of BCC, especially in resource-limited or remote areas. In such settings, deep learning algorithms can enhance screening efficiency and improve patient outcomes [39]. In addition, only the study by Zhu et al [22] included external validation. This limitation emphasizes the need for caution in interpreting the results and highlights the importance of future research focusing on the generalizability of deep learning models across different datasets and clinical environments. Another important point to note is that, although our results suggest superior performance on internal validation datasets compared to that of dermatologists, this performance does not necessarily translate well to external validation datasets [40,41]. Therefore, more external validations are essential to further confirm these findings and enhance the application of deep learning in dermatological diagnostics. Ultimately, claims of diagnostic superiority should be supported by prospective studies that better control for confounding variables and reflect real-world conditions [42]. Standardizing study design and outcome measures will also be crucial for improving the interpretability and comparability of future meta-analyses.

In addition to diagnostic performance, cost-effectiveness is a crucial factor for the widespread implementation of deep learning algorithms in routine clinical practice [43]. Unfortunately, our review did not identify any studies assessing the cost-effectiveness of deep learning algorithms in BCC diagnosis, which represents a significant gap that future research should address. However, studies in other medical fields such as ophthalmology and prostate cancer diagnosis have demonstrated the cost-effectiveness of AI technologies [43,44]. Trained and optimized AI models typically do not incur high maintenance costs while still providing valuable diagnostic data. These models could shorten diagnostic times, reduce treatment delays, and minimize unnecessary treatments, leading to substantial cost savings and improved patient care [43]. In summary, our findings suggest that, with further validation and improvement, deep learning algorithms could offer significant clinical benefits for BCC diagnosis.

### Limitations

When evaluating the results of this meta-analysis, it is essential to consider certain limitations. First, most of the included studies (14/15, 93%) were retrospective in design, with only the study by Wang et al [18] using a prospective design, which may introduce potential bias and confounding factors. Therefore, well-designed prospective studies are needed to validate the findings of this meta-analysis. Second, within the dermatoscopy-based deep learning algorithms, there were discrepancies in the definition of the gold standard for BCC diagnosis across the studies. Not all studies used histopathology as the gold standard, which may have a potential impact on diagnostic performance. However, we conducted a subgroup analysis on this variable, and the results showed no significant differences in sensitivity and specificity between different gold standards, suggesting that the conclusions were relatively robust. Third, most of the studies (7/15, 47%) relied on public datasets (such as the HAM10000 and International Skin Imaging Collaboration datasets), with fewer studies (3/15, 20%) using clinical dermatoscopy images from local hospitals for training and validation. This reliance on public datasets may limit the generalizability of the findings to real-world clinical settings. In addition, we only extracted the best-performing model from each study to avoid patient overlap as including multiple models from studies involving the same patients might distort the overall assessment. However, we acknowledge that this approach may carry the risk of overestimating the performance metrics. Future research should prioritize the evaluation of comparative performance among different algorithms to provide a more comprehensive understanding of their performance in clinical practice.

### Conclusions

This meta-analysis suggests that deep learning algorithms based on dermatoscopy exhibit strong diagnostic performance for detecting BCC. However, the retrospective design of many included studies and variations in RSs may restrict the generalizability of these findings. The models evaluated in the included studies generally showed improved performance over that of dermatologists in classifying dermatoscopic images of BCC using internal validation datasets, highlighting their potential to support future diagnoses. However, performance on internal validation datasets does not necessarily translate well to external validation datasets. Additional external validation of these results is necessary to enhance the application of deep learning in dermatological diagnostics.

## Appendix

Multimedia Appendix 1 Search strategy in PubMed, Embase, and Web of Science.

Multimedia Appendix 2 Diagnostic performance of included studies for dermatologists.

Multimedia Appendix 3 Dermoscopy technical details for the included studies.

Multimedia Appendix 4 Revised Quality Assessment of Diagnostic Accuracy Studies 2 tool for the included studies.

Multimedia Appendix 5 Subgroup analysis of deep learning algorithms performance in internal validation cohorts for basal cell carcinoma detection using dermoscopic images.

Multimedia Appendix 6 PRISMA checklist.

## Abbreviations

AI: artificial intelligence
AUC: area under the curve
BCC: basal cell carcinoma
CNN: convolutional neural network
FN: false negative
FP: false positive
MeSH: Medical Subject Headings
PRISMA: Preferred Reporting Items for Systematic Reviews and Meta-Analyses
PRISMA-DTA: Preferred Reporting Items for Systematic Reviews and Meta-Analyses extension for Diagnostic Test Accuracy
QUADAS-2: Quality Assessment of Diagnostic Accuracy Studies 2
ROC: receiver operating characteristic
RS: reference standard
TN: true negative
TP: true positive

## Data Availability

All data generated or analyzed during this study are included in this published article and its supplementary information files.
