# Supplementary material for: Deep Learning Algorithms in the Diagnosis of Basal Cell Carcinoma Using Dermatoscopy: Systematic Review and Meta-Analysis
Source: J Med Internet Res. 2025 Oct 3;27:e73541. doi: 10.2196/73541 (PMC12534767; doi:10.2196/73541)
Supplement: Multimedia Appendix 1 [file jmir_v27i1e73541_app1.docx]

Multimedia Appendix 1 Search strategy in PubMed, Embase and Web of Science.

| Database | Search strategy |
| --- | --- |
| PubMed | ("Artificial Intelligence"[Mesh] OR "Deep Learning"[Mesh] OR "Artificial Intelligence"[Title/Abstract] OR "AI"[Title/Abstract] OR "Deep Learning"[Title/Abstract] OR “Machine Intelligence”[Title/Abstract]) AND ("Skin Neoplasms"[Mesh] OR "Carcinoma, Basal Cell"[Mesh] OR “Skin Neoplasm”[Title/Abstract] OR “Cancer of Skin”[Title/Abstract] OR “Skin Cancer” [Title/Abstract] OR “Cancer of the Skin”[Title/Abstract] OR "basal cell carcinoma"[Title/Abstract] OR "intra-epithelial carcinoma"[Title/Abstract] OR "Bowen’s disease"[Title/Abstract] OR "actinic keratosis"[Title/Abstract] OR "non-melanoma skin cancer"[Title/Abstract]) AND ("dermoscopy"[MeSH] OR “epiluminescence microscopy”[Title/Abstract] OR "skin surface microscopy"[Title/Abstract] OR “dermoscopy”[Title/Abstract] OR “Dermoscopic”[Title/Abstract]) |
| Embase | ('artificial intelligence'/exp OR 'deep learning'/exp OR ‘Artificial Intelligence’:ab,ti OR ‘AI’:ab,ti OR ‘Deep Learning’:ab,ti OR ‘Radiomic’:ab,ti) AND ('skin tumor'/exp OR 'basal cell carcinoma'/exp OR 'non melanoma skin cancer'/exp OR ‘skin tumor’:ab,ti OR ‘Skin Neoplasm’:ab,ti OR ‘Cancer of Skin’:ab,ti OR “Skin Cancer’:ab,ti OR “Cancer of the Skin’:ab,ti OR 'basic cell carcinoma':ab,ti OR 'intra-epithelial carcinoma':ab,ti OR 'Bowen disease':ab,ti OR 'actinic keratosis':ab,ti OR 'non-melanoma skin cancer':ab,ti) AND ('dermatoscopy'/exp OR 'epiluminescence microscopy':ab,ti OR 'skin surface microscopy':ab,ti OR 'dermoscopy':ab,ti OR 'dermoscopic':ab,ti) |
| Web of Science | ((TS=("Artificial Intelligence" OR "Deep Learning" OR "AI" OR "Machine Intelligence")) AND TS=("Skin Neoplasms" OR "Carcinoma, Basal Cell" OR "Skin Neoplasm" OR "Cancer of Skin" OR "Skin Cancer" OR "Cancer of the Skin" OR "basal cell carcinoma" OR "intra-epithelial carcinoma" OR "Bowen's disease" OR "actinic keratosis" OR "non-melanoma skin cancer")) AND TS=("dermoscopy" OR "epiluminescence microscopy" OR "skin surface microscopy" OR "Dermoscopic") |
