# Supplementary material for: Deep Learning Algorithms in the Diagnosis of Basal Cell Carcinoma Using Dermatoscopy: Systematic Review and Meta-Analysis
Source: J Med Internet Res. 2025 Oct 3;27:e73541. doi: 10.2196/73541 (PMC12534767; doi:10.2196/73541)
Supplement: Multimedia Appendix 2 [file jmir_v27i1e73541_app2.docx]

**Multimedia Appendix 2 Diagnostic performance of included studies for dermatologists.**

| Author | Year | Number of total dermatologists | Training/junior dermatologists/senior dermatologists | Dermatologists | | | |
| --- | --- | --- | --- | --- | --- | --- | --- |
|  |  |  |  | TP | FP | FN | TN |
| Wang et al [18] | 2020 | 164 | NR/NR/NR | 8 | 2 | 2 | 58 |
| Zhu et al [22] | 2021 | 280 | NR/NR/NR | 20 | 6 | 5 | 169 |
| Maron et al [27] | 2019 | 112 | 4/67/28 | 44 | 5 | 16 | 235 |
| Minagawa et al [32]^a^ | 2020 | 30 | 10/NR/20 | 10 | 3 | 2 | 35 |
| Minagawa et al [32]^b^ | 2020 | 30 | 10/NR/20 | 7 | 0 | 5 | 38 |

TP true positive; TN true negative; FP false positive; FN false positive; NR not report; (a) Shinshu test set; (b) International Skin Imaging Collaboration test set.
