# Supplementary material for: Deep Learning Algorithms in the Diagnosis of Basal Cell Carcinoma Using Dermatoscopy: Systematic Review and Meta-Analysis
Source: J Med Internet Res. 2025 Oct 3;27:e73541. doi: 10.2196/73541 (PMC12534767; doi:10.2196/73541)
Supplement: Multimedia Appendix 3 [file jmir_v27i1e73541_app3.docx]

**Multimedia Appendix 3 Dermoscopy technical details for the included studies.**

| Author | Year | Polarized light dermascopy used | Contact/Non-Contact | Immersion Media Used |
| --- | --- | --- | --- | --- |
|  |  |  |  |  |
| Wang et al [18] | 2020 | NR | NR | NR |
| Kharazmi et al [19] | 2018 | Yes | Non-Contact | No |
| Maurya et al [20] | 2024 | Yes | Contact | No |
| Udriștoiu et al [21] | 2020 | NR | NR | NR |
| Zhu et al [22] | 2021 | NR | NR | NR |
| Serrano et al [23] | 2022 | NR | NR | NR |
| Cheng et al [24] | 2011 | NR | NR | NR |
| Maurya et al [25] | 2024 | Yes | Contact | No |
| Radhika and Chandana [26] | 2023 | Yes | Contact | No |
| Maron et al [27] | 2019 | NR | NR | NR |
| Naeem et al [28] | 2022 | Yes | Contact | No |
| Ali et al [29] | 2023 | NR | NR | NR |
| Panthakkan et al [30] | 2022 | NR | NR | NR |
| Priyeshkumar et al [31] | 2024 | NR | NR | NR |
| Minagawa et al [32] | 2020 | NR | NR | NR |

NR not report.
