# Supplementary material for: Deep Learning Algorithms in the Diagnosis of Basal Cell Carcinoma Using Dermatoscopy: Systematic Review and Meta-Analysis
Source: J Med Internet Res. 2025 Oct 3;27:e73541. doi: 10.2196/73541 (PMC12534767; doi:10.2196/73541)
Supplement: Multimedia Appendix 4 [file jmir_v27i1e73541_app4.docx]

**Multimedia Appendix 4 Revised QUADAS-2 tool for the included studies.**

| Author | Risk of bias | | | |  | Applicability concerns | | |
| --- | --- | --- | --- | --- | --- | --- | --- | --- |
|  | Patient selection ^a^ | Index test ^b^ | Reference standard ^c^ | Analysis ^d^ |  | Patient selection ^e^ | Index test ^f^ | Reference standard ^g^ |
| Wang et al 2020 [18] | L | L | L | L |  | L | L | L |
| Kharazmi et al 2018 [19] | U | L | L | L |  | L | L | L |
| Maurya et al 2024 [20] | U | L | L | L |  | L | L | L |
| Udriștoiu et al 2020 [21] | U | L | L | L |  | H | L | L |
| Zhu et al 2021 [22] | L | L | L | L |  | L | L | L |
| Serrano et al 2022 [23] | U | L | L | L |  | L | L | L |
| Cheng et al 2011 [24] | U | L | L | L |  | L | L | L |
| Maurya et al 2024 [25] | U | L | L | L |  | L | L | L |
| Radhika and Chandana 2023 [26] | U | L | L | L |  | H | L | L |
| Maron et al 2019 [27] | U | L | L | L |  | H | L | L |
| Naeem et al 2022 [28] | U | L | L | L |  | H | L | L |
| Ali et al 2023 [29] | U | L | L | L |  | H | L | L |
| Panthakkan et al 2022 [30] | U | L | L | L |  | H | L | L |
| Priyeshkumar et al 2024 [31] | U | L | L | L |  | H | L | L |
| Minagawa et al 2020 [32] | U | L | L | L |  | H | L | L |

L low; H high; U unclear.

a. **Patient selection**

- Low risk: No inappropriate exclusions.
- High risk: Inappropriate exclusions (e.g., excluding patients under 18, restricting to specific treatments/subtypes/timeframes). (For example, in this study, patients under 18 were excluded, only a specific type of BCC patients were included, only patients with a specific treatment were included, only patients with BCC in a specific location were included, or only patients who visited in the morning were included, etc.)
- Unclear: Insufficient information to assess exclusions.

b. **Index test**

- Low risk: Detailed model training/validation processes provided or cited from a prior publication with full modification details.
- High risk: Only model name reported without key training details (e.g., algorithm unspecified).
- Unclear: Model name given but training process indeterminable.

c. **Reference standard**

- Low risk: Final diagnosis made blinded to AI results.
- High risk: AI results used to inform final diagnosis.
- Unclear: Blinding status unreported.

d. **Analysis**

- Low risk: All enrolled participants included in meta-analysis.
- High risk: Selective exclusion of participants/subgroups.
- Unclear: Inclusion criteria inadequately described.

e. **Patient selection**

- Low risk: Study population aligns with meta-analysis inclusion criteria.
- High risk: Study includes ineligible patients per meta-analysis criteria. (For example, in this study, non-BCC patients were included.)
- Unclear: Population eligibility unclear.

f. **Index test**

- Low risk: AI definition matches meta-analysis criteria. (For example: It is an AI fully based on dermoscopic images for diagnosis.)
- High risk: AI definition partially deviates from criteria.
- Unclear: AI definition adequacy unverifiable.

g. **Reference standard**

- Low risk: Reference standard aligns with meta-analysis criteria.
- High risk: Reference standard inconsistently applied.
- Unclear: Reference standard details missing.
