# Supplementary material for: Deep Learning Algorithms in the Diagnosis of Basal Cell Carcinoma Using Dermatoscopy: Systematic Review and Meta-Analysis
Source: J Med Internet Res. 2025 Oct 3;27:e73541. doi: 10.2196/73541 (PMC12534767; doi:10.2196/73541)
Supplement: Multimedia Appendix 5 [file jmir_v27i1e73541_app5.docx]

## Multimedia Appendix 5 Subgroup analysis of deep learning algorithms performance in internal validation cohorts for basal cell carcinoma detection using dermoscopic images.

| Subgroup | Studies, n | Sensitivity(95%CI) | τ², I^2^ for sensitivity | Subgroup difference *P*-value | Specificity(95%CI) | τ², I^2^ for sensitivity | Subgroup difference *P*-value |
| --- | --- | --- | --- | --- | --- | --- | --- |
| AI method |  |  |  | 0.74 |  |  | 0.08 |
| CNN | 13 | 0.96 (0.92 - 0.98) | 1.29, 51.26% |  | 0.98 (0.97 - 0.99) | 0.48, 47.02% |  |
| Non-CNN | 3 | 0.95 (0.83 - 0.99) | 1.11, 79.56% |  | 0.95 (0.89 - 0.98) | 1.03, 89.53% |  |
| RS |  |  |  | 0.07 |  |  | 0.58 |
| Only Histopathology | 2 | 0.86 (0.62 - 0.96) | NA, NA |  | 0.97 (0.89 - 0.99) | NA, NA |  |
| Histopathology with expert consensus or clinical follow-up | 14 | 0.97 (0.94 - 0.98) | 1.13, 47.19% |  | 0.98 (0.96 - 0.99) | 0.84, 61.30% |  |
| Type of internal validation |  |  |  | 0.79 |  |  | 0.39 |
| Fold cross-validation | 3 | 0.97 (0.86 - 0.99) | 0.85, 30.44% |  | 0.99 (0.96 - 1.00) | 6.36, 12.09% |  |
| Random split test set | 10 | 0.96 (0.91 - 0.98) | 1.72, 54.89% |  | 0.98 (0.96 - 0.99) | 0.34, 43.76% |  |

AI artificial intelligence; CI Confidence Interval; CNN convolution neural network; RS reference standard.
